# Acute hyper-hypoxia accelerates the development of depression in mice via the IL-6/PGC1α/MFN2 signaling pathway

**DOI:** 10.1515/med-2024-1001

**Published:** 2024-08-09

**Authors:** Jialu Yu

**Affiliations:** Department of Anesthesiology, Guizhou Medical University, Guiyang, China; Department of Clinical Medicine, Guizhou Medical University, 14 Beijing Road, Guiyang 550000, China

**Keywords:** depression, interluekin-6/peroxisome proliferator-activated receptor gamma coactivator 1-alpha/mitofusin-2 signaling pathway, acute hypoxia, mice

## Abstract

**Background:**

Neural cell damage is an important cause of exacerbation of depression symptoms caused by hypoxia, but the mechanism behind it is still unclear. The purpose of this study is to elucidate the role of peroxisome proliferator-activated receptor gamma coactivator 1-alpha (PGC1α)/mitofusin-2 (MFN2) signaling axis in the development of depression in mice under hypoxia.

**Methods:**

Male Institute of Cancer Research mice (age, 6 weeks) were assigned to the normal group, chronic unpredictable mild stress group (CUMS group), or CUMS + hyper-hypoxia group (CUMS + H group). Mice in the CUMS and CUMS + H groups were exposed to CUMS for 28 days. Additionally, mice in the CUMS + H group were exposed to acute hyper-hypoxia from Day 21 for 7 days. After a total of 28 days, behavioral experiments were conducted. All mice were anesthetized and sacrificed. Levels of brain tissue interleukin (IL)-6, reactive oxygen species (ROS), adenosine triphosphate (ATP), and serotonin (5-HT) were analyzed.

**Results:**

As compared to the CUMS group, mice in the CUMS + H group had increased IL-6 and ROS levels, but lower open-field activity, preference for sucrose, hippocampal neuronal membrane potential, ATP, and 5-HT levels, as well as MFN2 and PGC1α levels.

**Conclusions:**

Acute hyper-hypoxia plays an important role in the development of depression via the IL-6/PGC1α/MFN2 signaling pathway.

## 1 Introduction

Depression is a mental illness and significant mental health concern characterized by low mood, decreased interest, and anhedonia that affects more than 350 million individuals worldwide [1,2]. The causes of depression are diverse and the pathogenesis is complex. However, relevant studies have found that the pathogenesis of depression is closely related to the hippocampus, which is a key brain region in the central nervous system responsible for learning, memory, and cognitive behavior [3]. At present, antidepressants, such as oral serotonin reuptake inhibitors, are mainly prescribed for clinical treatment of depression, although these drugs are limited by slow onset, many adverse reactions, and inconsistent efficacy [4].

Neural stem cells distributed in the dentate gyrus of the hippocampus have the potential for self-renewal, division, transformation into different types of brain cells and neurogenesis [5]. The monoamine neurotransmitters serotonin (5-HT), norepinephrine, and dopamine are mainly secreted in the brain and adrenal gland. Among these, 5-HT is as an important monoamine neurotransmitter in the central nervous system that directly or indirectly participates in regulation of human emotions, and acknowledged as an important biomarker of stress. Decreased levels of monoamine neurotransmitters in brain tissues have been linked to the incidence of depression [6,7]. Hypoxia alters metabolism of 5-HT in the brain and can activate the hypothalamic–pituitary–adrenal axis, which is the main regulator of stress responses. In response to stressors, the hypothalamus upregulates secretion of corticotropin-releasing hormone, which stimulates corticotropin production by the anterior pituitary, causing increased levels of corticosteroids, resulting in atrophy of hippocampal neurons, abnormal neuronal metabolism, destruction of neural plasticity, and ultimately the onset of depression.

In cellular respiration, glucose and oxygen react to produce adenosine triphosphate (ATP) on the inner membrane of mitochondria as a source of energy for cellular activities. A decrease in oxygen concentration can lead to lower energy production, as well as an imbalance in oxidative and antioxidant cellular responses, known as oxidative stress [8]. Low levels of ATP and oxygen inhibit normal functioning of the brain. The depression–oxidative stress hypothesis proposes that response to oxidative stress leads to excessive accumulation of reactive oxygen species (ROS) and consumption of antioxidants, which cause the destruction of the mitochondrial structure, mitochondrial homeostasis imbalance, and dysfunction of the mitochondrial residual respiratory chain via the peroxisome proliferator-activated receptor gamma coactivator 1-alpha (PGC1α)/mitofusin-2 (MFN2) signaling axis, leading to insufficient energy availability for nerve cells, which inhibits neurogenesis in the hippocampus [9–12]. Accumulated ROS can destroy highly sensitive cellular components, such as lipids and DNA, and trigger a series of inflammatory reactions through upregulated expression of inflammatory factors, resulting in damage to various organelles. Cytotoxic substances can damage the blood–brain barrier, promote the development of neuronal degenerative lesions, and reduce neuroplasticity [13].

The aims of this study were to investigate the effects of hypoxia on the development of depression in mice and to clarify the involvement of the PGC1α/MFN2 signaling axis.

## 2 Materials and methods

### 2.1 Study approval

The study protocol was approved by the Institutional Animal Care and Use Committee of Guizhou Medical University (approval no. 320928240100005531) and conducted in accordance with the Guide for the Care and Use of Laboratory Animals (https://www.ncbi.nlm.nih.gov/books/NBK54050/).

### 2.2 Experimental animals and grouping

Male Institute of Cancer Research mice (animal batch number: 320928240100005531; *n* = 30; age, 6 weeks; mean body weight [BW], 25 ± 2 g) were purchased from Zhuhai Baitantong Biotechnology Co., Ltd (Zhuhai, Guangdong Province, China) and randomly allocated to one of the three groups (*n* = 10 each): a normal group, chronic unpredictable mild stress group (CUMS group), or CUMS + hypoxia group (CUMS + H group). All mice were acclimated for 14 days prior to the experiment in an animal care facility with *ad libitum* access to feed and water.

### 2.3 Establishment of a mouse model of depression

**Figure 1:**
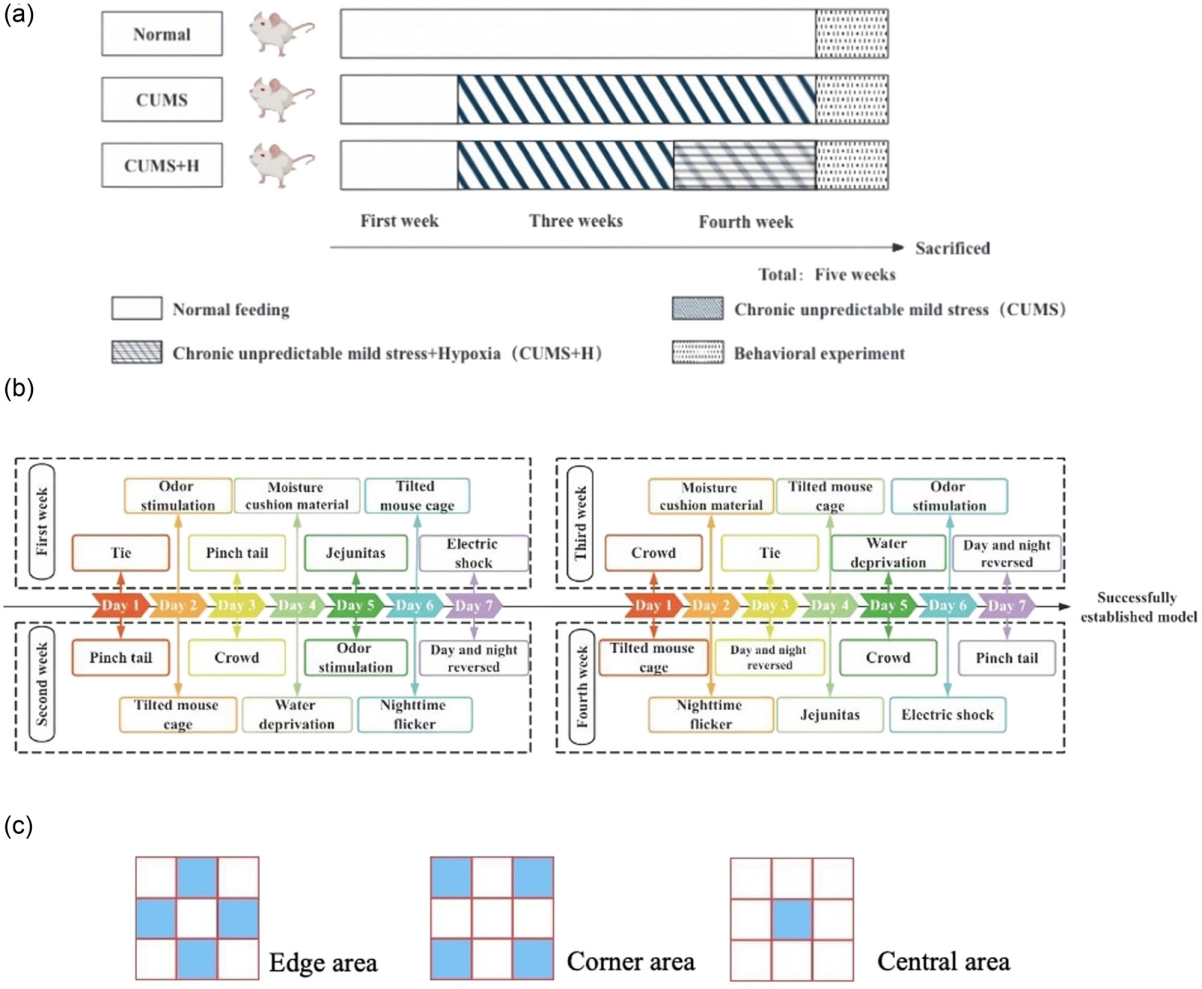
Establishment of a mouse model of depression. (a) Mice were subjected to CUMS for 4 weeks along with acute hyper-hypoxia intervention in the CUMS + H group. (b) Mice were subjected to CUMS for 4 weeks. (c) Specific composition of the box used for the open field test.

Mice in the CUMS group were kept in single cages and stimulated for 14 consecutive days by tail pinch (3 min), jejunitas (1 day), water deprivation (1 day), tie (3 h), day and night reversal (1 day), electric shock (30 V, 5 s, 5 s intervals, total of 300 s), odor stimulation (3 h), moisture cushion material (1 day), crowding (5 mice/cage, 1 day), nighttime flicker (12 h), or tilting of the cage (45°, 12 h) (Figure 1).

Mice in the CUMS + H group were subjected to the same conditions as those in the CUMS group and then subjected to hypoxic conditions for 7 days in an incubator with an oxygen concentration of 15 ± 1%. Meanwhile, the mice in the CUMS group were continuously exposed to CUMS for 7 additional days.

### 2.4 BW measurements

The BW of each mouse was measured three times per week and recorded. Changes to the mean BW were compared among the groups.

### 2.5 Sucrose preference test

The mice were acclimated in single cages with *ad libitum* access to feed and water for 3 days. On Day 4, each mouse was given a bottle of pure water and a second bottle of water with 1% sucrose. The position of each bottle was changed every 12 h but at the same height. The drinking bottle was weighed before the experiment and after 24 h. The preference for water with sucrose was calculated as sugar water consumption/(pure water consumption + sugar water consumption) × 100%.

### 2.6 Open field test

Before starting the open field test, the mice were put in a dark room (room temperature, 20°C) for at least 30 min to prevent stress in response to a new environment. The box used for the open field test was divided into nine areas measuring 70 × 70 cm. After 30 min, each mouse was gently placed in the center of the open field box in a quiet environment and activities over a period of 5 min were recorded with VisuTrack rodent behavior analysis software (ver. 3.0.01; ViewPoint Behavior Technology, Lyon, France). After each mouse was recorded, the feces and urine were removed, and the open field was cleaned with 75% alcohol and dried with a dry cloth before the next mouse was tested. The horizontal movement score was calculated by counting the number of horizontal lines that the mouse had crossed, where each was assigned a value of 1. A vertical movement was considered to have occurred when both front legs of the mouse were off the ground or on the sidewall of the open field box. The time of vertical movements was calculated as the vertical movement score. The scores of horizontal and vertical movements were calculated for each group (Figure 1c).

### 2.7 Sample processing

At the end of the behavioral experiment, each mouse was anesthetized by intraperitoneal injection of tribromoethanol (Avodine®; Avonic Life Sciences, Ambala Sadar, India). Then, the thoracic cavity was dissected, an intravenous indwelling needle was inserted into the left ventricle, the right atrial appendage was resected, and 50 mL of phosphate-buffered saline (PBS) was perfused followed by 50 mL of 4% paraformaldehyde. Afterward, the whole brain was carefully removed.

### 2.8 Enzyme-linked immunosorbent assay (ELISA)

Serum samples and the whole brain were collected from each mouse and homogenated. The supernatant was collected for analysis of interleukin (IL)-6 and ROS in the serum samples in addition to ATP and 5-HT levels in the whole brain samples with the use of commercial ELISA kits (Huainan Lianke Biological Medicine Co., Ltd, Huainan, China) and an automated immunoassay analyzer (RT-6000; Rayto Life and Analytical Sciences Co., Ltd, Shenzhen, China). The serum levels of IL-6 and ROS in addition to whole brain levels of ATP and 5-HT were calculated against standard curves.

### 2.9 Mitochondrial membrane potential (MMP) assay

Hippocampal tissues were rinsed twice with cooled anatomical fluid and the meninges were dissected. Then, the tissues were digested with trypsin, mixed using a micropipette, transferred to new tubes, diluted to the desired concentration with culture medium, and transferred to a polylysine-treated flat dish or cover glass for isolation and culture of hippocampal neuronal cells. The isolated cells were added to the wells of six-well culture plates and the culture solution was removed. Afterward, the cells were washed once with PBS, mixed with 1 mL of cell culture solution and 1 mL of JC-1 dyeing solution (Beyotime Institute of Biotechnology, Shanghai, China), and incubated for 20 min at 37°C in a cell incubator. During incubation, the prepared JC-1 dye buffer (1×) was cooled in an ice bath. After incubation, the supernatant was removed and each well was washed twice with JC-1 dye buffer (1×). Following the addition of 2 mL of cell culture solution, the cells were imaged with an EVOS™ M5000 Imaging System (Thermo Fisher Scientific, Waltham, MA, USA).

### 2.10 Polymerase chain reaction (PCR) analysis of MFN2 and PGC1α mRNA levels in hippocampal tissues

The hippocampal tissues were digested and centrifuged at 12,000 rpm for 10 min. The RNA concentration and purity were measured using the Bioanalyzer RNA 6000 Nano assay (Agilent Technologies, Inc., Santa Clara, CA, USA). The mRNA was reverse transcribed into complementary DNA using a commercial kit (Vazyme Biotech Co., Ltd, Nanjing, China) with gene-specific primers (MFN2: 5′-TCC AAC CCC TGC TTG ACA G-3′/5′-TGA ACG CTG TCA CCT CAA CC-3′; PGC1α:5′-TCT CTA GGT ACC GTC TTG AG-3′/5′-CAC ACA CAC ACA CAA AGC AAC-3′; PGC1α: 5′-TCT CTA GGT ACC GTC TTG AG-3′/5′-CAC ACA CAC ACA CAA AGC AAC-3′) and a CFX96 Touch™ Real-Time PCR Detection System (Bio-Rad Laboratories, Hercules, CA, USA). The relative expression levels of the genes of interest were calculated using the 2^−ΔΔCt^ method against glyceraldehyde 3-phosphate dehydrogenase as an internal control.

### 2.11 Immunohistochemical analysis of MFN2 and PGC1α protein levels in hippocampal tissues

Paraffin-embedded sections of hippocampal tissues were dewaxed, incubated with 3% H_2_O_2_ at room temperature for 30 min to block endogenous peroxidase activity, and incubated overnight at 4°C with primary antibodies against MFN2 (dilution ratio, 1:300) and PGC1α (dilution, 1:500), followed by horseradish peroxidase-conjugated secondary antibodies (dilution, 1:2,000) at 37°C for 30 min. Afterward, the tissues were washed three times for 5 min with PBS. The nuclei were stained with hematoxylin (Sigma-Aldrich Corporation, St. Louis, MO, USA) for 3–10 min, followed by 1% hydrochloric acid, and rinsed with water, water with 1% ammonia, and water again. Finally, the slices were dehydrated, clarified, sealed with neutral gum, and imaged under a microscope.

### 2.12 Statistical analysis

The data were analyzed and plotted using Prism 9 software (ver. 9.4.0; GraphPad Software, Inc., San Diego, CA, USA). The data are presented as the mean ± standard deviation. The *t*-test was used for comparison between two groups and one-way analysis of variance for comparisons among three or more. A probability (*p*) value < 0.05 was considered statistically significant.

## 3 Results

### 3.1 Correlation between BW loss and behavioral score

**Figure 2:**
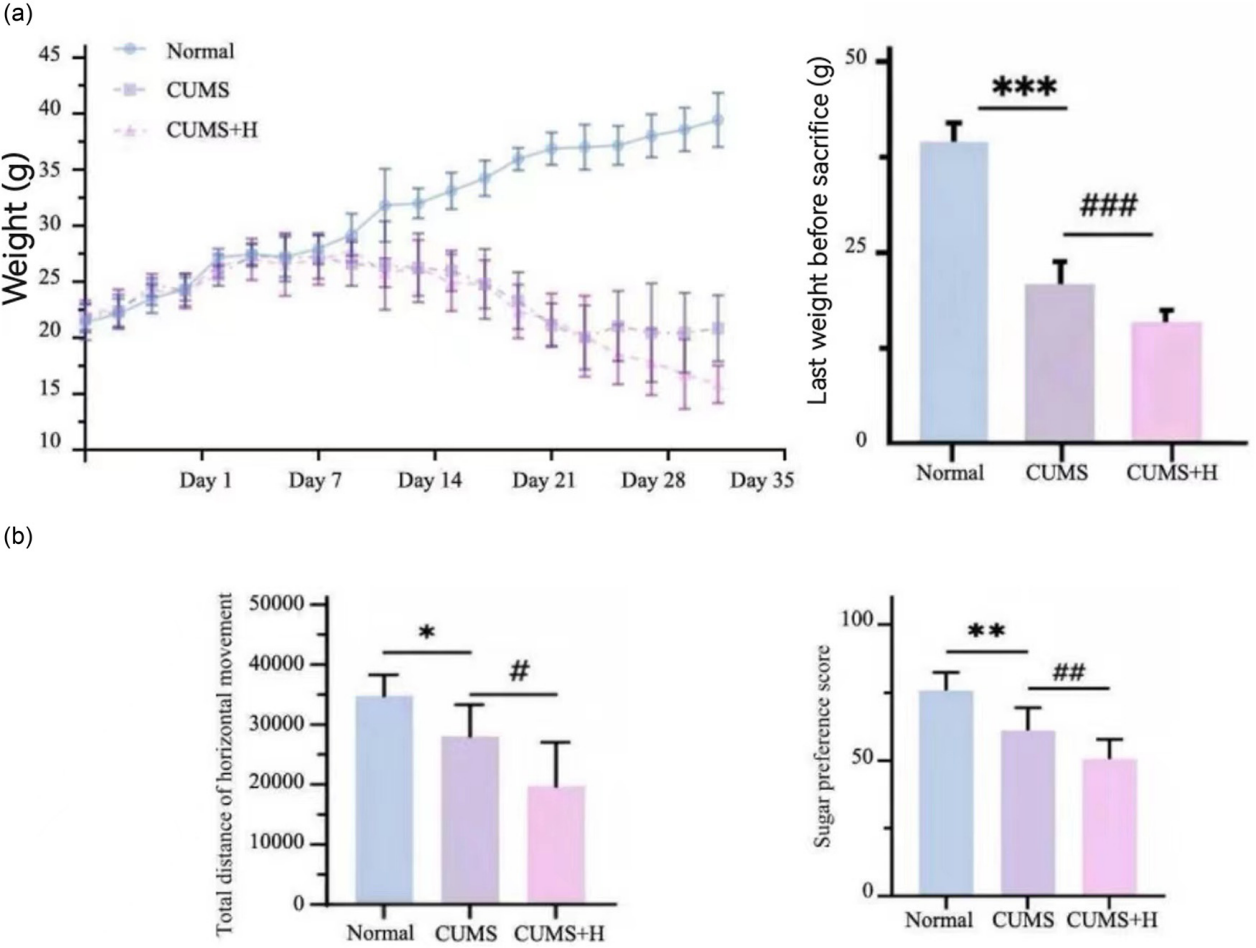
Mouse BW and behavioral scores. (a) Changes to the BW of mice. During the modeling period, the mean BW of the CUMS group remained unchanged, while that of the CUMS + H group had decreased. As indicated in the bar chart, the mean BW of the CUMS group decreased by about half (***p < 0.001), while the CUMS + H group had the lightest BW (^###^ *p* < 0.001). (b) Change to the preference of mice for sugar and the open field test score. As compared to the normal group, the preference for sucrose and the score of open field test had decreased in the CUMS group (**p < 0.01, *p < 0.01) and further decreased in the CUMS + H group (^##^ *p* < 0.05, ^#^ *p* < 0.05).

The mean BW of mice in the normal group gradually increased, but there was no significant difference between the CUMS and normal groups on Day 16. However, after Day 16, the mean BW of mice in the CUMS group slowly decreased by 48.7% as compared to the normal group (Figure 2a). Meanwhile, before establishing the model, behavioral analysis showed that there was no significant difference in the preference for water with 1% sucrose among the groups (mean score = 74.36 ± 1.52%). As compared to the normal group, the preference for water with 1% sucrose was 14.7% lower in the CUMS group.

The results of the open field test showed that the number of times the mice entered the central area and the total distance of horizontal movement had decreased by about 51.1% in the CUMSs group as compared to the normal group, demonstrating symptoms of depression, such as decreased interest and willingness to explore new environments. Collectively, these results indicate successful establishment of a mouse model of depression that was suitable for the hypoxia intervention experiments.

The preference of mice for water with 1% sucrose was 10.6% lower in the CUMS + H group as compared to the CUMS group. The number of times that mice entered the central area of the open field and the total distance of horizontal movement decreased by 25.0% in the CUMS + H group as compared to the CUMS group. These findings indicate that acute hypoxia worsened symptoms of depression (Figure 2b).

### 3.2 CUMS increased serum levels of IL-6 and ROS, as well as decreased the whole brain tissue levels of ATP and 5-HT

**Figure 3:**
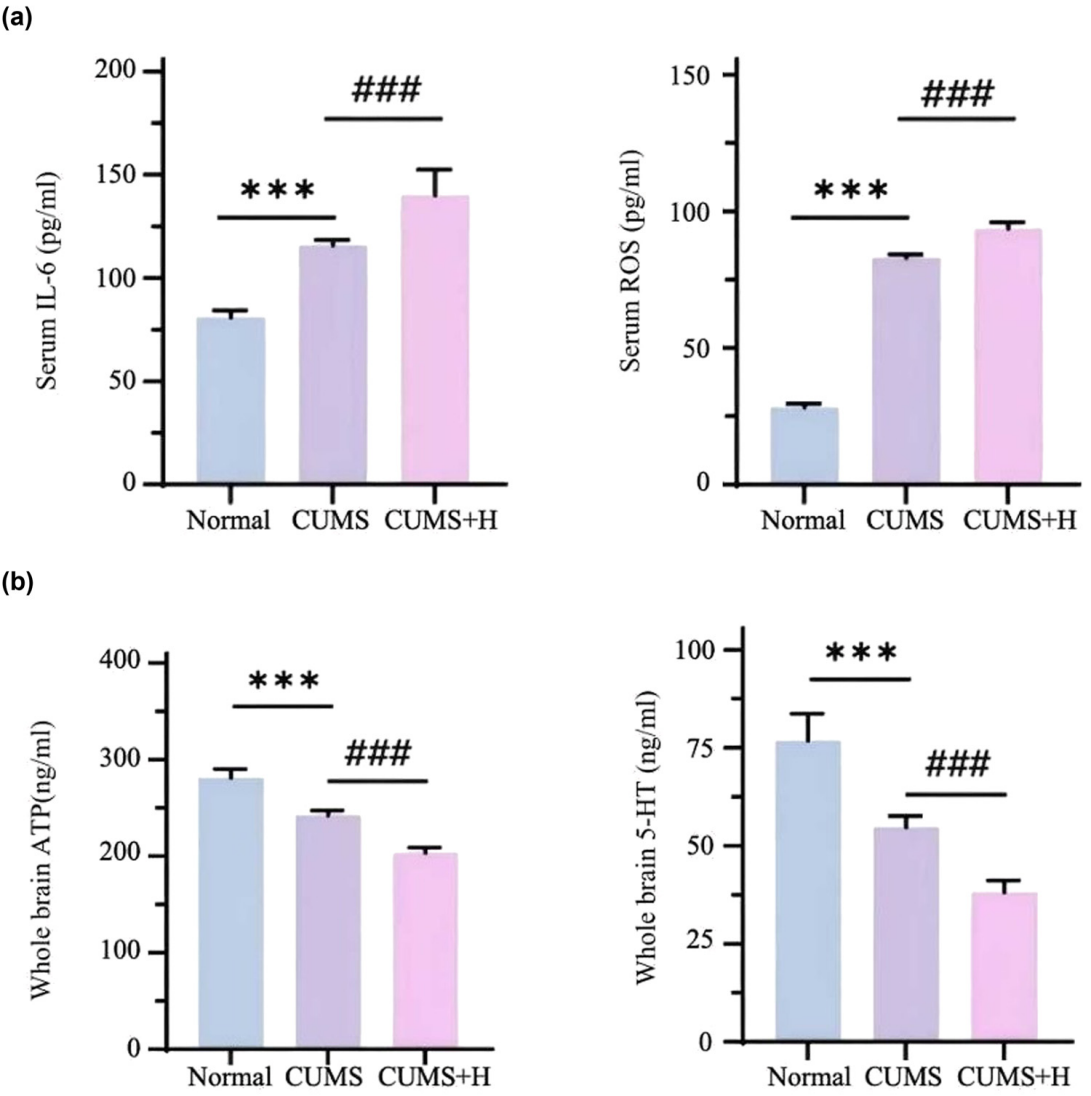
Serum levels of IL-6 and ROS and whole brain tissue levels of ATP and 5-HT. (a) Serum levels of IL-6 and ROS were significantly higher in the CUMS group than the normal group (***p < 0.001) and further increased in the CUMS + H group (***p < 0.001, ^###^ *p* < 0.05). (b) As com*p*ared to the normal group, whole brain tissues levels of ATP and 5-HT were significantly lower in the CUMS group (***p < 0.001) and further decreased in the CUMS + H group (^###^ *p* < 0.001).

Serum levels of IL-6 and ROS were increased by 2- and 3-fold in the CUMS group as compared to the normal group. As compared to the CUMS group, serum levels of IL-6 and ROS were increased by 35.2 and 20.1%, respectively, in the CUMS + H group. Inflammation increased accumulation of peroxides, which reduced energy production and growth of hippocampal neurons (Figure 3a). Meanwhile, as compared to the normal group, hippocampal tissue levels of 5-HT and ATP decreased by 37.3 and 16.4%, respectively, in the CUMS group and further decreased by approximately 49.0 and 18.4% in the CUMS + H group (Figure 3b). The hypothalamic–pituitary–adrenal axis is activated by a decrease in 5-HT content, which disrupts hormone levels and leads to neuronal atrophy in the brain. Acute hypoxia exacerbates inflammation, imbalance of oxidative and antioxidant responses, and reduces monoamine neurotransmitter levels, which worsen symptoms of depression.

### 3.3 Changes to MMP in mouse hippocampal neurons

**Figure 4:**
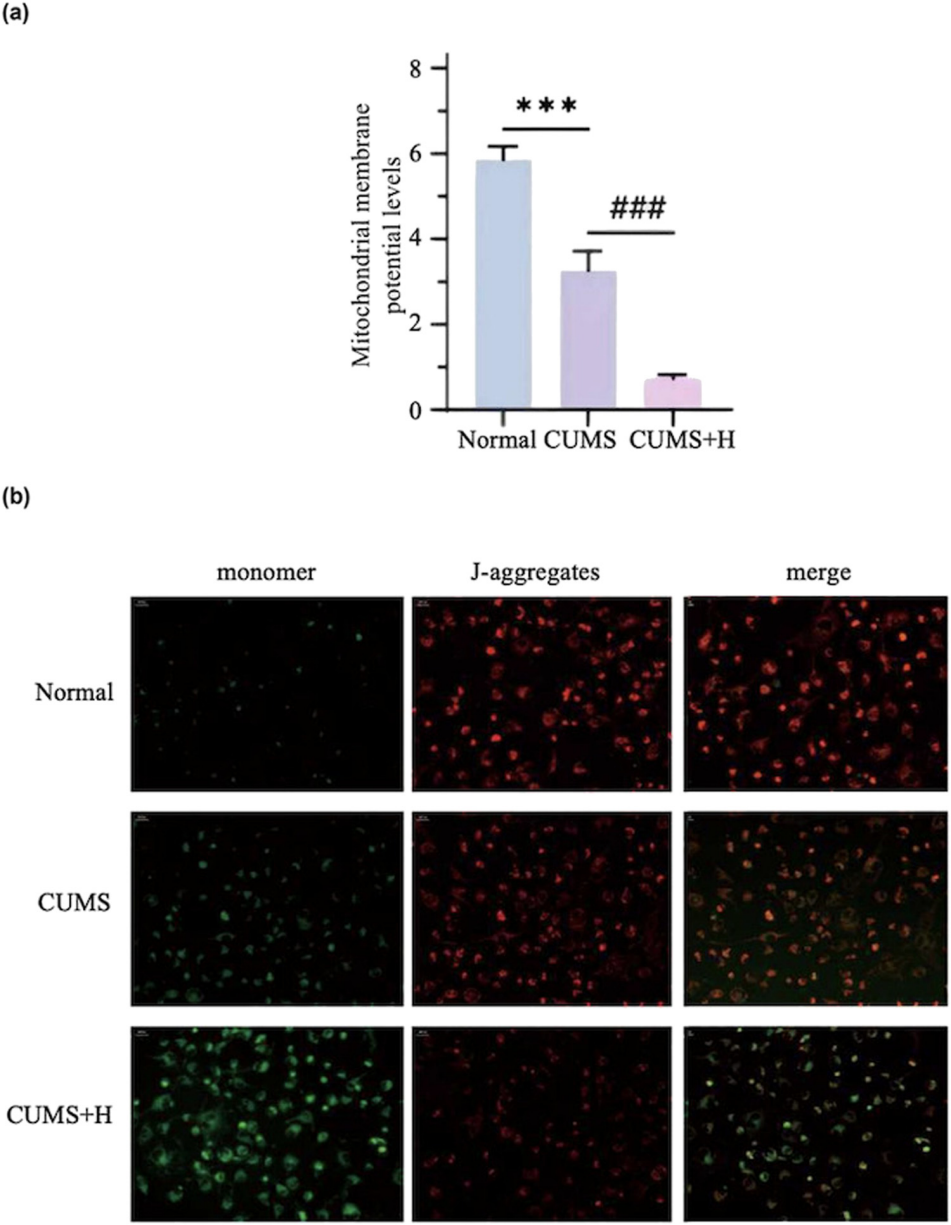
Detection of MMP. Red fluorescence is indicative of healthy mitochondria, while green fluorescence is indicative of unhealthy mitochondria. As compared to the normal group, MMP was significantly lower in the CUMS group (***p < 0.001) and further decreased in the CUMS + H group (^###^ *p* < 0.001).

The MMP was about 38.7% lower in the CUMS group than the normal group and further decreased in the CUMS + H group, accounting for only 15.4% of the CUMS group (Figure 4a), demonstrating that mitochondrial damage in the hippocampus is associated with the occurrence of depression. Under acute hyper-hypoxia conditions, mitochondrial damage was more extensive in the hippocampus of depressed mice, which was accompanied by respiratory chain rupture, incomplete oxidative responses, excessive accumulation of toxic products, insufficient brain energy production, neuronal damage, and the occurrence and development of depression (Figure 4b).

### 3.4 CUMS decreased expression of MFN2 and PGC1α in mouse hippocampal tissues

**Figure 5:**
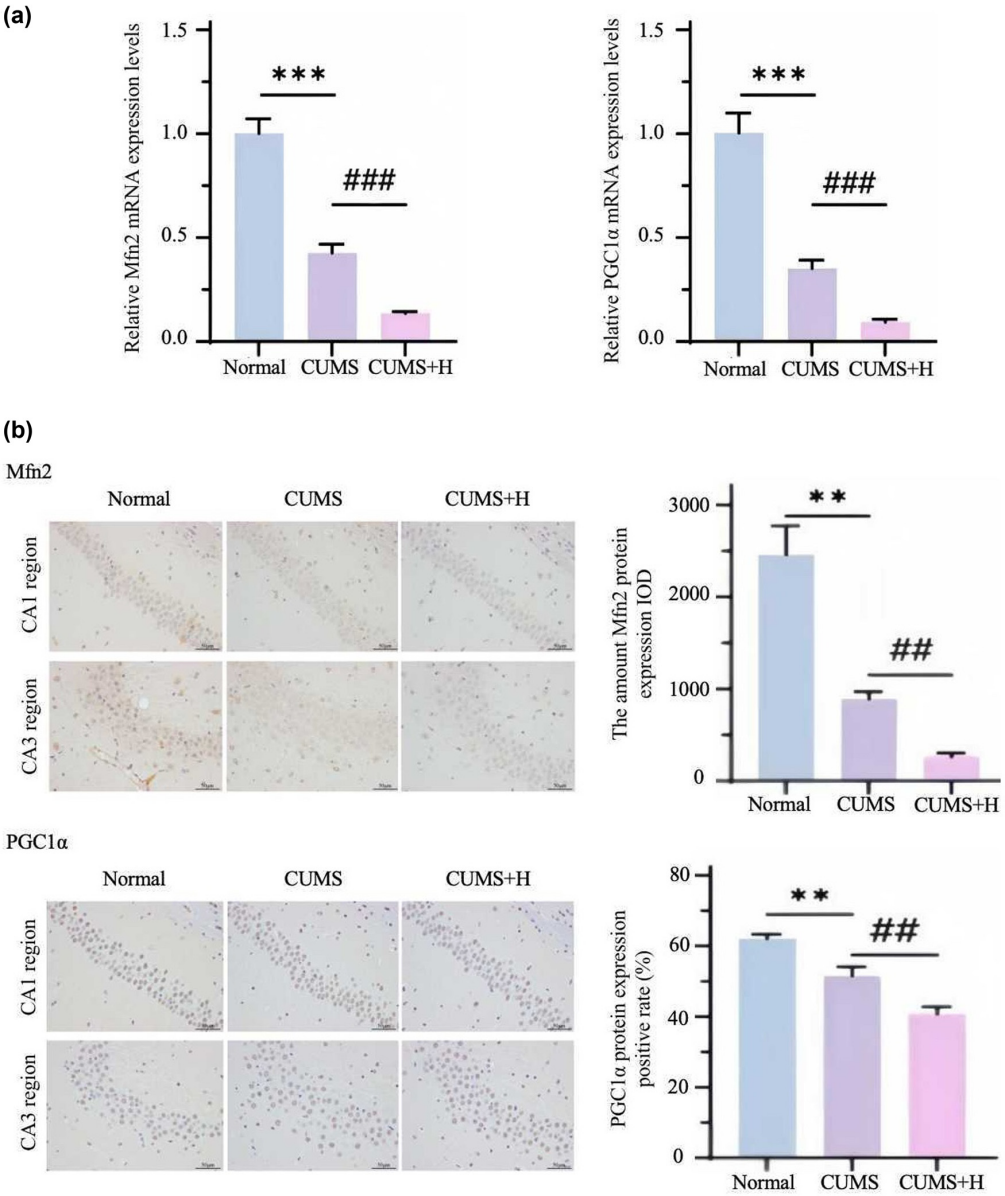
Changes to hippocampal levels of PGC1α and MFN2. (a) The mRNA expression levels of MFN2 and PGC1α in the hippocampus were significantly lower in the CUMS group than the normal group (***p < 0.001) and further decreased in the CUMS + H group (^###^ *p* < 0.001). (b) As compared to the normal group, the protein levels of MFN2 and PGC1α were significantly decreased in the CUMS group (**p < 0.01, ^###^ *p* < 0.001) and further decreased in the CUMS + H group (**p < 0.01, ^##^ *p* < 0.01).

PCR and immunohistochemical analyses were used to detect the mRNA and protein levels, respectively, of MFN2 and PGC1α in the hippocampal tissues of mice. As compared to the normal group, the mRNA levels of MFN2 and PGC1α in the hippocampal tissues decreased by 57.2 and 65.0%, respectively, in the CUMS group and reduced by about 29.8% in the CUMS + H group (Figure 5a). Meanwhile, changes to the protein levels of MFN2 and PGC1α in the hippocampal tissues were consistent with the mRNA levels. The integrated optical density levels of MFN2 and PGC1α protein in hippocampal tissues decreased by 500 and 10%, respectively, in the CUMS + H group as compared to the CUMS group (Figure 5b). Reduced expression of PGC1α and MFN2 leads to mitochondrial fragmentation, excessive division, fusion disorders, disruption of the respiratory chain, reduced energy production, neurogenesis disorders, and insufficient production of monoamine neurotransmitters.

## 4 Discussion

Depression is a psychiatric disorder with a wide range of causes. Although the pathogenesis remains unclear, hypoxia reportedly worsens the symptoms of depression. Appropriate oxygen levels are crucial to maintain health and prevent various diseases, especially psychiatric and neurological disorders [14–16]. Studies have confirmed that hypoxia caused by rapid advancement at high altitudes can lead to impaired memory and discrimination, but no relevant animal experiments have been conducted to explain the reasons and mechanisms [17]. In this study, a mouse model of depression induced by acute hyper-hypoxia was successfully established. As compared to the CUMS group, BW gain rate, sugar preference, and the open field test score decreased in the CUMS + H group, suggesting that acute hyper-hypoxia aggravates symptoms of depression. Furthermore, serum levels of ROS were significantly increased in the CUMS + H group along with disruption of the respiratory chain and mitochondrial damage, which caused hypoxia and insufficient energy availability in the hippocampal tissues. According to the monoamine neurotransmitter hypothesis, hypoxia leads to a gradual decrease in 5-HT levels in brain tissues, which aggravates the symptoms of depression. The results of the present study showed that levels of 5-HT and ATP in hippocampal tissues were further decreased in the CUMS + H group, demonstrating further aggravation of the symptoms of depression, which was consistent with the findings of previous studies [18–20]. Meanwhile, serum levels of IL-6 were significantly increased in the CUMS + H group. IL-6 activates the metabolic enzyme indoleamine 2,3-dioxygenase and significantly reduces synthesis of 5-HT, indicating that inflammatory cytokines play an important role in brain regions closely related to depression and 5-HT conversion [21,22].

Since mitochondrial function is very important to brain function and is easily damaged by hypoxia, the expression profiles of two molecules related to mitochondrial function, PGC1α and MFN2, were explored in this study. It has been reported that serum levels of PGC1α are downregulated in patients with depression. Meanwhile, MFN2, a protein present in the mitochondrial membrane that controls mitochondrial fusion and metabolism in mammalian cells, is downregulated in the brain tissues of mice with glucocorticoid-induced depression [23–25]. These results suggest that impaired mitochondrial biogenesis and fusion due to low expression of PGC1α and MFN2 are linked to the pathogenesis of depression [26–28]. Also, acute hyper-hypoxia reduced protein levels of PGC1α and MFN2 RNA in the hippocampal tissues of mice, which confirmed that acute hyper-hypoxia can aggravate the symptoms of depression via a mechanism that is closely related to changes in PGC1α and MFN2 contents. The pathogenesis of depression has been linked to molecular abnormalities in the brain, but social, environmental, and inflammatory factors, in addition to neurogenesis, are also involved in the occurrence and development of depression [29].

Based on the IL-6-PGC1α-MFN2 pathway, this study explored the correlation between acute hyper-hypoxia and the occurrence and development of depression, which concluded that acute hyper-hypoxia aggravates the symptoms of depression. However, it remains unclear whether reduced gene expression under non-hypoxic conditions would achieve the same effect. Hence, we plan to further explore this hypothesis in future animal experiments. Based on the findings of the present study and previous research, improving the hypoxic state of nerve cells or slowing mitochondrial damage may be an effective strategy for the treatment of depression in the future [30,31]. Hyperbaric oxygen has been reported to reduce depression-like behavior and neuroinflammation in rats with traumatic brain injury, nourish nerve cells, slow cell and mitochondrial damage, and relieve symptoms of depression [32–34]. At low pO_2_ levels, mitochondria-targeted antioxidants improve myofilament Ca2^+^ sensitivity, increase PGC1α induction, and protect mitochondria, thereby improving symptoms of depression [35,36].

In conclusion, acute hyper-hypoxia can regulate the IL-6-PGC1α-MFN2 signaling pathway, aggravate inflammation, influence mitochondrial biogenesis and fusion, damage hippocampal neurons, reduce cognition, memory, and emotional regulation, and accelerate the occurrence and development of depression. Hence, improving cell hypoxia or slowing mitochondrial damage has potential as a new clinical intervention for depression.
